# Effect of Gender, Rearing, and Cooking on the Metabolomic Profile of Porcine Muscles

**DOI:** 10.3390/metabo10010010

**Published:** 2019-12-22

**Authors:** Shoko Sawano, Keishi Oza, Tetsuya Murakami, Mako Nakamura, Ryuichi Tatsumi, Wataru Mizunoya

**Affiliations:** 1 Department of Food and Life Science, School of Life and Environmental Science, Azabu University, Sagamihara 252-5201, Japan; 2 Department of Bioresource Sciences, Faculty of Agriculture, Kyushu University, Fukuoka 819-0395, Japan; 3 Fukuoka Agriculture and Forestry Research Center, Chikushino 818-0004, Japan; 4 Department of Animal Science and Biotechnology, School of Veterinary Medicine, Azabu University, Sagamihara 252-5201, Japan

**Keywords:** pork, meat, skeletal muscle, fiber type, cooking

## Abstract

To clarify the relationship between the fiber type composition and meat quality, we performed metabolomic analysis using porcine longissimus dorsi (LD) muscles. In the LD of pigs raised outdoors, the expression of myosin heavy chain (MyHC)1 (slow-twitch fiber marker protein) was significantly increased compared with that of MyHC1 in pigs raised in an indoor pen, suggesting that rearing outdoors could be considered as an exercise treatment. These LD samples were subjected to metabolomic analysis for examining the profile of most primary and secondary metabolites. We found that the sex of the animal and exercise stimulation had a strong influence on the metabolomic profile in the porcine skeletal muscles, and this difference in the metabolomic profile is likely in part due to the changes in the muscle fiber type. We also examined the effects of cooking (70 °C for 1 h). The effect of exercise on the metabolomic profile was also maintained in the cooked muscle tissues. Cooking treatment resulted in an increase in some of the metabolite levels while decreasing in some other metabolite levels. Thus, our study could indicate the effect of the sex of the animal, exercise stimulus, and cooking on the metabolomic profile of pork meat.

## 1. Introduction

Free amino acids stimulate taste and can modify the palatability of foods depending on the concentrations at which they are present in the foods [1,2,3,4]. To date, free amino acids are considered as the most important taste components of meat. In addition to free amino acids, purine nucleotides, such as inosine monophosphate (IMP), are also thought to be important components of taste in meat, thereby considerably potentiating the taste responses of free amino acids [5]. The presence of IMP suggests the existence of taste modifiers, as well as the tastants in food and the complexity in the induction of the taste stimuli. Although these free amino acids and purine nucleotides are undoubtedly important components of meat taste, we think there are more substances that affect meat taste and flavor. For example, taurine was not thought to be a taste substance because it is tasteless; however, taurine has been reported to show a positive correlation with umami intensity and is thought to impart full-bodied taste in meat taste or flavor by an unknown mechanism [6]. Thus, metabolomic substances as well as taurine could have the potential to affect meat taste and flavor. One-by-one analysis of each substance contained in meat will take a long time, but it is possible to depict the whole metabolite profiling of meat as the combination and association of these metabolites form the overall taste and flavor.

Metabolomics in food analysis is the study of the metabolites present in the foods to describe and predict the properties of the food palatability and taste. Analytical methods have been developed to elucidate the profile of the components responsible for the overall food taste and flavor. For example, metabolomic analysis is performed for the metabolite profiling of strawberries and for identifying fruit attributes influencing hedonics and sensory perception based on consumer ratings [7]. A few metabolomic applications have been reported in meat. Metabolomic analysis has been performed to clarify the sensory characteristics and flavor of beef [8,9], the effect of finishing forage on beef [10], the effect of storage condition of ground beef [11], and the key metabolites during the postmortem aging of beef [12] or pork [13]. The approach to find a novel biomarker that indicates meat quality in relation to the water-holding capacity has also been reported [14]. However, more analyses need to be performed to clarify the relationship between various meat characteristics and metabolites.

Skeletal muscle tissues are composed of slow-twitch (type 1) and fast-twitch (type 2) muscle fibers. Metabolically, slow-twitch fibers have abundant mitochondria and myoglobin and rely on oxidative metabolism, whereas fast-twitch fibers have less mitochondria and myoglobin and mainly rely on the glycolytic pathway. In addition to these metabolic traits, the muscle fiber composition affects various meat properties, such as the color, pH, water-holding capacity, tenderness, and nutritional value of meat [15]. In our previous study, we confirmed that there was a strong positive correlation between myosin heavy chain (MyHC)1 (slow-twitch fiber marker protein) composition and total free amino acid concentrations in bovine muscles, thereby suggesting that a high content of slow-twitch fibers contributes to the intense flavor of meat derived from amino acids [16]. In fact, in a tasting panel evaluation of lamb, which is a red meat rich in slow-twitch fibers, the lamb meat was classed as having a more intense flavor than white meat, which is assumed to be rich in fast-twitch fibers [17].

Thus, we thought that meat rich in slow-twitch fibers may have a different composition of metabolites from that in the fast-twitch predominant meat and this composition may not consist of only free amino acids. The aim of this study was to provide an insight into the profiling of pork meat metabolites under two conditions known to affect muscle fiber types: sex of the animal and exercise treatment. In humans, the proportion of slow-twitch fibers in women has been reported to be higher than that in men [18,19,20]. This greater proportion of the slow-twitch fibers in women could partially explain their higher oxidative capacity. The adaptive benefits of exercise training are commonly attributed to the fast-to-slow fiber type transition and increased mitochondrial energetic capacity [21]. Moreover, we also examined the alterations in the metabolite profiling induced by the cooking of pork meat. Although the sample size in this study was limited, the obtained results were remarkable, and these findings will contribute to the progress in the analysis of taste and flavor of meat in the future.

## 2. Results and Discussion

**Figure 1:**
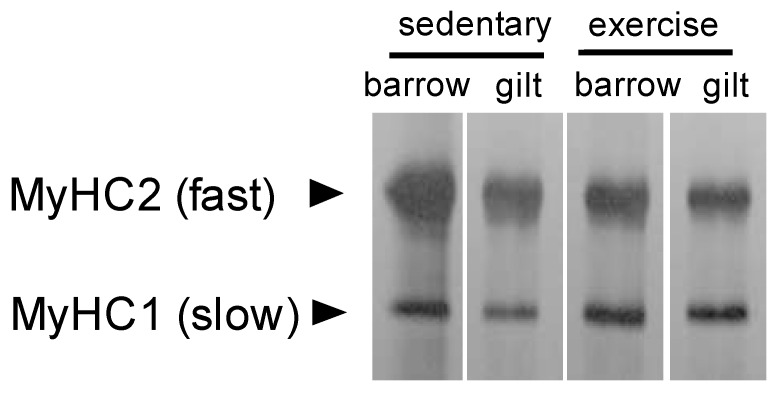
Separation of myosin heavy chain (MyHC) isoforms of the porcine longissimus dorsi (LD) muscles from sedentary or exercising barrows and gilts by using SDS-PAGE.

First, we measured the muscle fiber type compositions in the longissimus dorsi (LD) muscle tissue samples from barrows (castrated males) and gilts (females), which were raised indoors (sedentary) and outdoors (exercising animals). MyHC1 (slow-twitch fiber marker) and MyHC2 (fast-twitch fiber marker) isoform compositions were measured by SDS-PAGE. The percentage of MyHC1 isoforms in the barrows and gilts was similar but it was slightly higher in the gilts (18.0% vs. 22.3% in sedentary pigs, 30.6% vs. 33.4% in exercising pigs) (Figure 1). We found that the exercise treatment increased the slow-type MyHC1 composition significantly in the outdoor animal group of the barrow and gilt compared to that in the sedentary group of the barrow and gilt (20.2 ± 2.2% vs. 32.0 ± 1.4%, *p* < 0.05 by *t*-test, *n* = 2 for each group). It is well-known that prolonged exercise training could induce fast-to-slow fiber type transition. In this study, the pigs reared outdoors exercised voluntarily. Voluntary wheel running has been widely used as a model of non-interventional exercise training that induces adaptive changes in the skeletal muscle fiber type from fast-twitch to slow-twitch fibers. More specifically, the fiber type transition among fast-twitch subtypes (types 2A, 2X, and 2B) is commonly induced by endurance exercise; shift from type 2B/2X toward type 2X/2A. It is reported that voluntary wheel running induced fiber type transition of 2B/2X-to-2A in mice [22] or 2B-to-2A/2X in rats [23]. However, a shift from type 2-to-type 1 fibers may occur in limited muscle tissues under longer duration, higher volume endurance type events. For example, voluntary wheel running for four weeks induced type 2-to-type 1 fiber type conversion in rat plantaris muscle but not in soleus muscle [24]. Similarly, type 2-to-type 1 fiber type conversion in pigs also occurred during voluntary exercise although the muscle tissue was LD in our experiment. In this study, the proportion of slow-twitch fibers was slightly higher in gilts, which is in accordance with that reported previously in humans, although the effects of castration on the porcine muscle fiber type have still not been elucidated.

**Figure 2:**
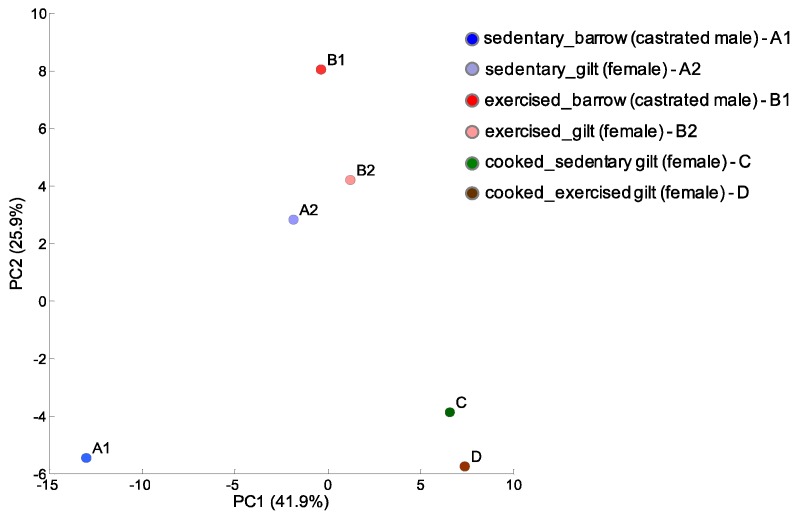
Principal component analysis (PCA) of the porcine longissimus dorsi (LD) muscle metabolomic profiles of sedentary or exercising barrows and gilts. We also analyzed cooked gilt LD muscles.

In this study, we performed global CE-TOFMS analysis to target metabolites involved in primary and secondary metabolism, such as sugars, amino acids, nucleotides, and other ionic metabolites in porcine LD muscles of sedentary barrow and gilt, exercising barrow and gilt, and cooked gilts. In this analysis, 130 peaks were detected. The analyzed metabolite profile of the LD muscles, i.e., pork loin, showed notable changes in each experimental condition: sex, exercise, and cooking. A principle component analysis (PCA) was performed to visualize the condition-related effects on the metabolites in LD muscles (Figure 2). Overall, the PCA demonstrated clear clustering in sex and cooking conditions. The peaks of the exercising barrow and gilt showed a slight separation from each other but they were still clustered together. Surprisingly, the separation was profound between the sedentary barrow and gilt, suggesting that the exercise stimulus decreased the sex differences seen in sedentary animals. Exercise is known to increase oxidative capacity. Hence, the alteration of oxidative capacity might be attenuated in gilts because females have higher oxidative capacity intrinsically.

**Figure 3:**
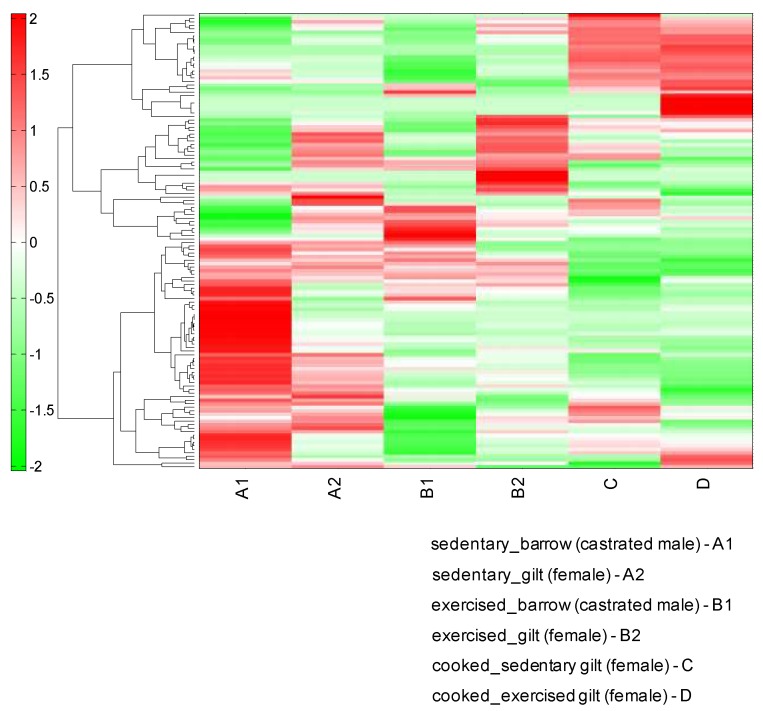
Hierarchical clustering analysis (HCA) of the porcine longissimus dorsi (LD) muscle metabolomic profiles of sedentary or exercising barrows and gilts. We also analyzed cooked gilt LD muscles.

The hierarchical clustering analysis (HCA) of the metabolites in the LD muscles showed a remarkably different pattern in the different experimental conditions (Figure 3). The results of the HCA classification were very similar to those of the PCA analysis. The sedentary barrow sample showed a different metabolite pattern from that of the sedentary gilt muscles or the exercising barrow muscle.

Table 1 shows the changes in the metabolite ratios under the three different conditions. There was a notable increase in the sugars of fructose-6-phosphate and glucose-6-phosphate in the meat of the sedentary gilt compared to that in the meat of the sedentary barrow. Interestingly, the levels of these two sugars were also markedly increased by the exercise stimulus. The ratio of the gilt/barrow and exercise/sedentary seems to have common metabolic features. Since both conditions are known to show higher oxidative metabolism, the substrates in the glycolytic pathways might be spared. The exercise did not evoke marked alterations in the metabolites in gilts, while the metabolites in barrows showed various differences due to exercise. These findings indicate that there may be sex differences in the response to exercise.

As expected, the cooked LD muscles contained a completely different profile of the substances compared with that in the respective uncooked muscles. Even after the cooking treatment, there were still clear differences between the sedentary and exercised samples. In fact, the differences in the profile of the metabolites in the uncooked muscles was maintained even after heating at 70 °C for 1 h. Cooking treatment increased the levels of N-acetylornithine, ribulose 5-phosphate, N-acetyl lysine, N-acetylneuraminic acid, ATP, methionine sulfoxide, methionine, phenylalanine, tryptophan, and tyrosine compared with those in the four uncooked samples. In particular, ATP, N-acetylneuraminic acid, and methionine sulfoxide were detected only in the cooked samples, thereby suggesting that these substances are unique in cooked meat. It was interesting to note that ATP emerged after cooking because ATP is thought to be an unstable molecule. Conversely, the cooking treatment decreased the levels of diethanolamine, isoglutamic acid, succinic acid, glutamine, reduced glutathione, thiamine phosphate, gamma-aminobutyric acid, ethanolamine, putrescine, and ethanolamine phosphate compared with those in the four uncooked samples. In particular, thiamine phosphate, gamma-aminobutyric acid, and putrescine were not detected in the cooked samples, thereby suggesting that these substances were degraded or metabolized by cooking.

The levels of volatile aroma compounds were not measured in this study because the adopted CE-TOFMS is not optimized for the identification of these compounds. Flavor is an important factor for determining the palatability of food and the taste formed by the cooking of food. For example, the sugars in the meat are likely the precursors of the flavor components produced by the Maillard reaction, which is one of the most important pathways occurring primarily between the amino acids and the reducing monosaccharides for flavor formation in cooked foods [25]. Meinert et al. reported that glucose and glucose-6-phosphate increased the flavor-related volatile components more than ribose and ribose 5-phosphate in minced pork [26]. In our analysis, lipids were not targeted, although they are responsible for the volatile components of the meat flavor after cooking [27]. It should be emphasized that a comprehensive analysis of the volatile aroma compounds responsible for the flavor of meat would contribute to the understanding of whole meat palatability in the future.

The sex of the animal and exercise stimulation influence the type of metabolites contained in porcine skeletal muscles. The metabolic profile of sedentary gilt was already similar to exercised barrow even though MyHC1 expressions were increased in both the gilt and barrow. We thought that the regulation of metabolism and that of MyHC expression are independent although these two factors are closely related. In fact, adaptations in muscle metabolic capacity to prolonged exercise training can occur without fiber type alterations [28]. Thus, we assumed the change in the metabolic profile was partly attributed to the changes in the muscle fiber type, and partly attributed to other factors such as upregulation of metabolic enzyme expression. The effect of exercise is also maintained in the cooked muscle tissues. It is necessary to verify whether the changes observed in this study were not just individual differences. In the future, we will try to elucidate the relationship between metabolite profiles and taste (flavor) of meat by integrating sensory evaluation and metabolome analysis.

## 3. Materials and Methods

### 3.1. Meat Sample

The pork loins of Large White were obtained from a local meat shop. The pigs were reared in a farm located in the Fukuoka Agriculture and Forestry Research Center (Chikushino, Japan). The pigs were slaughtered in an approved slaughterhouse and dressed according to Japanese standard commercial procedures, then distributed to the meat shop. We chose the entire pork loins from one barrow and one gilt raised indoors in a 1.9 m × 3.5 m (6.7 m²) pen or raised outdoors where they were allowed to graze over a total area of 369 m² (12.3 m × 30 m) during daytime for 6 h (9:30 to 15:30) for 32 days in finishing period. From the entire pork loins chilled for 120 h since slaughter (normal storage period in Japan), we excised the LD muscles. The excised middle LD muscle blocks were vacuum packed and frozen at −30 °C until preparation. We used the single sample for each treatment (*n* = 1).

### 3.2. Sample Preparation

Muscle samples were thawed overnight at 4 °C. The small portion of the middle LD muscle tissue was ground to powder with a mortar and pestle, cooled with liquid nitrogen, and stored at −20 °C for metabolomic analysis or at −80 °C for protein assay. For obtaining cooked samples, the small block (about 4 g) of thawed LD was vacuum packed and heated in a water bath at 70 °C for 1 h and cooled under running tap water for 30 min. The cooked muscle tissue was snap frozen in liquid nitrogen and ground to powder with a mortar and pestle, cooled with liquid nitrogen, and stored at −20 °C until metabolomic analysis.

### 3.3. MyHC Isoform Content Determination

A motor-driven small pestle was used to homogenize each thawed uncooked sample of the muscle (~50 mg) in an SDS solution (10% SDS, 40 mM DTT, 5 mM EDTA, and 0.1 M Tris-HCl buffer (pH 8.0)) on ice. The SDS solution contained the Protease Inhibitor Cocktail for Use with Mammalian Cell and Tissue Extracts (Nacalai Tesque, Inc., Kyoto, Japan) in a 1:100 ratio. The sample homogenates were heated in boiling water for 3 min. The total protein concentrations were assayed using the Pierce BCA Protein Assay Kit (Thermo Fisher Scientific, Waltham, MA, USA), with bovine serum albumin as the standard. The samples were diluted in 2× sample buffer (100 mM DTT, 4.0% SDS, 0.16 M Tris-HCl (pH 6.8), 43% glycerol, and 0.2% bromophenol blue) and dH_2_O to give final protein concentrations of 20 ng/μL in 1× sample buffer. These protein samples were subjected to high-resolution SDS-polyacrylamide gel electrophoresis for assessing the MyHC isoform composition, as described in detail previously [29]. The gel contained 8% acrylamide (acrylamide/bisacrylamide ratio = 99:1) and 35% (*v*/*v*) glycerol. After loading the samples (100 ng protein), electrophoresis was performed at a constant voltage of 140 V for 22 h at 4 °C. The gels were stained with Silver Stain Kanto III (Kanto Chemical Co. Inc., Tokyo, Japan) and dried. The bands were captured on an imager (Fusion SL-4, Vilber Lourmat), and the relative contents of the MyHC isoforms were quantified by densitometry using the ImageJ 1.34s software (Rasband W, National Institutes of Health, Bethesda, MD, USA). MyHC isoforms were identified according to their different migration rates (MyHC1 > 2).

### 3.4. Sample Pretreatment for Metabolome Analysis

Metabolome measurements were performed through a facility service at Human Metabolome Technologies (HMT) Inc., Tsuruoka, Japan. Briefly, approximately 30 mg of frozen uncooked or cooked LD samples was plunged into 1200 μL of 50% acetonitrile/Milli-Q water containing internal standards (Solution ID: 304-1002, Human Metabolome Technologies, Inc., Tsuruoka, Japan) at 0 °C to inactivate the enzymes. The tissue was homogenized 4 times at 1500 rpm for 120 s by using a tissue homogenizer and then the homogenate was centrifuged at 2300× *g* at 4 °C for 5 min. Subsequently, 400 μL of the upper aqueous layer was centrifugally filtered through a Millipore 5-kDa cutoff filter at 9100× *g* at 4 °C for 120 min to remove the proteins. The filtrate was centrifugally concentrated and re-suspended in 50 μL of Milli-Q water for CE-MS analysis.

### 3.5. CE-TOFMS Analysis

CE-TOFMS was performed using an Agilent CE Capillary Electrophoresis System equipped with an Agilent 6210 Time of Flight mass spectrometer, Agilent 1100 isocratic HPLC pump, Agilent G1603A CE-MS adapter kit, and Agilent G1607A CE-ESI-MS sprayer kit (Agilent Technologies, Waldbronn, Germany). The systems were controlled by the Agilent G2201AA ChemStation software version B.03.01 for CE (Agilent Technologies, Waldbronn, Germany). The metabolites were analyzed using a fused silica capillary (50 μm i.d. × 80 cm total length), with a commercial electrophoresis buffer (Solution ID: H3301-1001 for cation analysis and H3302-1021 for anion analysis, HMT) as the electrolyte. The sample was injected at a pressure of 50 mbar for 10 s (approximately 10 nL) for the cation analysis and 25 s (approximately 25 nL) for the anion analysis. Spectrometry was performed by scanning from *m*/*z* 50 to 1000. Other conditions were as described previously [30].

### 3.6. Data Analysis

Peaks were extracted using the automatic integration software MasterHands (Keio University, Tsuruoka, Japan) in order to obtain peak information, including *m*/*z*, migration time for CE-TOFMS measurement (MT), and peak area [31]. Signal peaks corresponding to isotopomers, adduct ions, and other product ions of known metabolites were excluded, and the remaining peaks were annotated with putative metabolites from the HMT metabolite database based on their MTs and *m*/*z* values determined by TOFMS. The tolerance range for the peak annotation was configured at ±0.5 min for MT and ±10 ppm for *m*/*z*. In addition, peak areas were normalized against those of the internal standards and then the resultant relative area values were further normalized by the sample amount. HCA and PCA were performed using HMT’s proprietary software, PeakStat and SampleStat, respectively. The detected metabolites were plotted on metabolic pathway maps by using the VANTED (Visualization and Analysis of Networks containing Experimental Data) software [32].

### 3.7. Statistics

Results of MyHC analysis are expressed as means ± SE. We used a two-tailed *t*-test calculated by Excel 2016 for Mac (Microsoft), and significance was set at *p* < 0.05.

## Figures and Tables

**Table 1:** Metabolite ratios in the three different conditions. Numbers in the red boxes are a ratio of >2 and those in blue boxes are a ratio of <0.5. The intensity of the color corresponds to the magnitude of the ratio <0.1 or >10.

Compound Name	Gilt/Barrow		Exercise/Sedentary		Cooked/Uncooked	
	Sedentary	Exercise	Barrow	Gilt	Sedentary	Exercise
1-Methylhistidine, 3-Methylhistidine	0.59	1.09	0.80	1.46	0.64	0.64
2-(Creatinine-3-yl) propionic acid	N.D.	N.D.	N.D.	N.D.	N.D.	N/A
2-Aminoisobutyric acid, 2-Aminobutyric acid	0.78	1.10	0.58	0.81	0.65	0.73
2-Hydroxybutyric acid	1.10	zero	zero	zero	0.79	N.D.
2-Hydroxyvaleric acid	2.38	0.67	1.76	0.49	0.87	0.96
3-Hydroxybutyric acid	1.28	1.90	0.92	1.37	1.00	0.73
3-Methyladenine	N/A	1.25	N/A	1.05	zero	zero
4-Methylpyrazole	1.18	zero	0.89	zero	zero	N/A
5’-Deoxy-5’-methylthioadenosine	N.D.	N.D.	N.D.	N.D.	N.D.	N/A
5-Oxoproline	0.71	1.10	0.42	0.65	0.67	2.39
Adenosine	N.D.	N.D.	N/A	N.D.	N.D.	N/A
ADMA	0.96	1.22	0.63	0.81	zero	1.67
ADP	0.93	zero	3.11	zero	3.96	N/A
ADP-ribose	N/A	0.66	N/A	0.84	0.40	0.53
Ala	0.73	0.84	0.73	0.84	0.70	0.82
AMP	0.93	0.10	14.49	1.63	7.84	5.90
Anserine_divalent	1.03	1.60	0.83	1.29	0.72	0.63
Arg	0.84	1.42	0.60	1.01	1.28	1.38
Argininosuccinic acid	zero	N.D.	zero	N.D.	N.D.	N.D.
Asn	0.72	1.39	0.51	0.99	1.09	1.15
Asp	0.45	0.93	0.58	1.20	1.05	1.07
ATP	N.D.	N.D.	N.D.	N.D.	N/A	N/A
Betaine	1.33	1.44	0.96	1.04	0.90	0.76
Butyrylcarnitine	0.36	1.49	0.19	0.79	0.67	0.74
Carnitine	0.94	1.24	0.67	0.88	0.72	0.63
Carnosine	1.33	1.26	1.01	0.96	0.96	0.84
Choline	0.43	0.87	0.35	0.70	0.40	0.48
Citrulline	0.71	1.26	0.43	0.76	0.74	0.65
Creatine	1.06	1.13	0.91	0.98	0.91	0.78
Creatinine	1.33	1.34	0.82	0.82	1.25	3.68
Cys	4.65	1.53	3.22	1.06	1.47	1.02
Cysteine glutathione disulfide	0.21	N/A	zero	0.56	zero	0.55
Cystine	0.06	N.D.	zero	zero	zero	N.D.
Cytidine	1.05	1.12	0.78	0.84	0.83	0.96
Daminozide Ala-Ala	N/A	N/A	N.D.	1.14	1.90	1.42
Diethanolamine	1.06	zero	1.24	zero	zero	N.D.
Dyphylline	2.69	0.83	2.88	0.89	0.99	0.90
Ethanolamine	0.91	1.01	0.87	0.97	0.56	0.50
Ethanolamine phosphate	0.80	0.84	1.04	1.09	zero	0.68
Fructose 6-phosphate	36.20	0.42	69.38	0.80	1.55	0.98
GABA	1.13	1.16	0.76	0.78	zero	zero
Gln	0.87	0.77	0.88	0.78	0.50	0.66
Glu	0.62	1.79	0.36	1.04	1.06	0.94
Glu-Glu	2.20	1.90	1.38	1.19	1.27	1.00
Gluconic acid	2.48	2.10	1.35	1.14	0.88	0.49
Gluconolactone	N/A	1.50	N/A	1.15	1.14	zero
Glucose 1-phosphate	N/A	0.51	N/A	0.85	1.15	0.72
Glucose 6-phosphate	43.39	0.39	83.94	0.76	0.81	0.54
Glutathione (GSH)	0.97	0.89	1.09	1.00	0.78	0.65
Glutathione (GSSG)_divalent	0.25	0.81	0.21	0.68	0.17	0.26
Gly	0.83	1.14	0.63	0.87	0.75	0.75
Gly-Asp	zero	N.D.	zero	N.D.	N.D.	N/A
Gly-Gly	N.D.	N/A	N.D.	N/A	N.D.	zero
Gly-Leu	N.D.	N.D.	N.D.	N.D.	N/A	N.D.
Glyceric acid	N/A	2.59	N/A	0.81	0.40	0.65
Glycerol	1.34	0.63	1.87	0.88	0.94	0.95
Glycerol 3-phosphate	0.97	1.24	0.57	0.73	0.51	0.93
Glycerophosphocholine	1.00	0.64	1.58	1.01	0.61	0.71
GMP	1.12	1.30	1.05	1.22	1.06	0.89
Guanine	0.45	1.03	0.35	0.80	0.74	1.13
Guanosine	0.91	0.84	0.76	0.70	0.93	1.04
His	0.73	1.15	0.62	0.99	1.06	0.89
His-Glu	N.D.	N.D.	N.D.	N.D.	N.D.	N/A
Homocarnosine	1.41	1.28	1.18	1.07	0.94	0.81
Hydroxyproline	0.79	0.96	0.62	0.76	0.48	0.70
Hypotaurine	0.43	0.70	0.66	1.06	0.46	0.60
Hypoxanthine	0.46	1.15	0.33	0.83	1.00	1.23
Ile	0.84	1.24	0.60	0.88	1.31	1.47
IMP	1.50	1.63	1.03	1.12	0.94	0.93
Inosine	1.24	1.02	1.03	0.85	0.97	0.98
Isobutyric acid Butyric acid	0.84	N/A	zero	1.46	zero	zero
Isoglutamic acid	0.71	zero	0.41	zero	zero	N.D.
Lactic acid	1.43	1.23	1.14	0.98	0.82	0.77
Leu	0.86	1.43	0.59	0.99	1.38	1.39
Lys	0.74	1.38	0.53	0.99	1.18	1.26
Malic acid	0.46	1.63	0.34	1.20	0.76	0.41
Malonylcarnitine	N/A	N.D.	N.D.	zero	zero	N.D.
Met	1.07	1.62	0.63	0.96	1.81	1.83
Methionine sulfoxide	N.D.	N.D.	N.D.	N.D.	N/A	N/A
*myo*-Inositol 1-phosphate *myo*-Inositol 3-phosphate	N.D.	N/A	N.D.	N.D.	N/A	1.00
*N*-Acetyllysine	1.81	1.57	1.20	1.04	1.95	1.88
*N*-Acetylneuraminic acid	N.D.	N.D.	N.D.	N.D.	N/A	N/A
*N*-Acetylornithine	1.43	2.00	0.86	1.20	2.11	1.82
*N*-Methylalanine	0.76	1.22	0.56	0.89	0.57	0.60
*N*^5^-Ethylglutamine	0.44	0.94	0.65	1.39	0.72	0.75
*N*^6^,*N*^6^,*N*^6^-Trimethyllysine	1.80	1.21	1.92	1.29	0.74	0.65
*N*^6^-Methyllysine	0.66	0.73	1.04	1.15	1.00	0.95
NADH	0.89	2.43	0.28	0.76	1.21	1.02
Nicotinamide	2.27	0.89	2.16	0.85	0.79	1.05
*O*-Acetylcarnitine	0.83	1.72	0.32	0.67	1.43	1.51
*O*-Acetylhomoserine 2-Aminoadipic acid	0.73	0.88	0.57	0.69	0.50	0.69
Ornithine	0.89	2.10	0.40	0.94	0.76	0.79
Pantothenic acid	0.71	1.20	0.57	0.96	zero	0.81
Phe	1.01	1.27	0.71	0.90	1.54	1.62
Phosphorylcholine	0.36	0.77	0.45	0.98	0.81	1.09
Pro	0.68	1.05	0.53	0.82	0.75	0.84
Putrescine	0.91	0.99	1.00	1.09	zero	zero
Ribulose 5-phosphate	1.40	1.34	1.07	1.03	1.43	1.45
*S*-Adenosylhomocysteine	1.07	0.84	1.26	1.00	1.16	1.35
*S*-Adenosylmethionine	1.45	1.12	1.38	1.06	0.59	0.67
*S*-Methylcysteine	0.90	1.43	0.79	1.25	0.87	0.57
Saccharopine	N/A	0.70	N/A	1.17	0.75	0.60
Sedoheptulose 7-phosphate	zero	zero	0.40	N.D.	N.D.	N.D.
Ser	0.68	1.34	0.51	1.01	1.14	1.09
Spermidine	0.82	1.01	0.69	0.85	0.66	0.86
Spermine	2.92	1.35	1.82	0.84	1.08	1.14
Stachydrine	1.52	N/A	zero	0.78	0.83	0.91
Succinic acid	0.67	0.62	0.89	0.82	0.54	0.76
Taurine	0.50	0.89	0.61	1.08	0.51	0.67
Terephthalic acid	N.D.	N.D.	N.D.	N.D.	N.D.	N/A
Thiamine	1.08	1.72	0.52	0.84	1.04	1.00
Thiamine phosphate	1.31	1.17	1.21	1.09	zero	zero
Thr	0.69	1.35	0.50	0.98	1.07	1.03
Thr-Asp Ser-Glu	1.10	N.D.	zero	zero	1.52	N/A
Trigonelline	1.08	1.58	0.59	0.86	0.98	0.70
Trp	0.92	1.24	0.74	0.99	1.32	1.36
Tyr	0.92	1.60	0.60	1.05	1.50	1.55
UDP-glucose UDP-galactose	N.D.	zero	N.D.	N.D.	N.D.	N.D.
UDP-*N*-acetylgalactosamine UDP-*N*-acetylglucosamine	N.D.	N/A	N.D.	N/A	N.D.	zero
UMP	1.77	1.97	1.11	1.24	0.99	0.74
Urea	0.99	0.91	0.73	0.67	0.76	0.76
Uridine	1.14	0.85	0.97	0.73	0.83	0.99
Val	0.74	1.24	0.55	0.92	1.09	1.14
β-Ala	0.89	0.96	0.76	0.82	0.82	0.64
β-Ala-Lys	1.98	1.44	1.41	1.02	0.66	0.74
γ-Butyrobetaine	1.06	1.44	0.61	0.83	0.81	0.76
γ-Glu-Cys	N.D.	N/A	N.D.	N/A	N.D.	zero

N.D. denotes it could not be detected in both conditions. N/A denotes it could not be calculated due to “the division by zero”. The “zero” denotes its numerator was not detected.
